# A novel approach to monitoring selective fatigue in female softball athletes: evaluating load-velocity relationship variables and specific performance metrics

**DOI:** 10.3389/fphys.2025.1702738

**Published:** 2026-01-09

**Authors:** Hongzhen Zhang, Zhaoqian Li, Qiuyu Yu, Zan Cheng, Xiaoqing Wang, Xing Zhang, Amador García-Ramos, Danica Janicijevic

**Affiliations:** 1 School of Physical Education, Shandong University, Jinan, China; 2 Beijing Lucheng Sports Technology School, Beijing, China; 3 Department of Physical Education and Sport, Faculty of Sport Sciences, University of Granada, Granada, Spain; 4 Department of Sports Sciences and Physical Conditioning, Faculty of Education, Universidad Católica de la Santísima Concepción, Concepción, Chile; 5 Faculty of Sports Science, Ningbo University, Ningbo, China; 6 Department of Radiology, Ningbo No. 2 Hospital, Ningbo, China

**Keywords:** bench press, blood lactate, physical education, rating of perceived exertion, resistance training, velocity loss

## Abstract

**Introduction:**

The purpose of this study was to assess whether bench press load-velocity (L-V) relationship variables serving as indicators of maximal theoretical force capacity (*L*_0_), maximal theoretical velocity capacity (*v*_*0*_), and maximal theoretical power capacity (A_line_), as well as the softball-specific performance metrics (hit and throw distance), could be used to effectively monitor the selective fatigue induced by two different bench press training protocols.

**Methods:**

The bench press L-V relationship variables and softball-specific performance metrics of 12 professional female softball players were measured on three separate occasions: (I) following passive rest (non-fatigue condition), (II) after light-load ballistic bench press throw (LLB), and (III) after heavy-load traditional bench press (HLT). Additionally, blood lactate and rating of perceived exertion (RPE) were assessed after LLB and HLT training protocols.

**Results:**

A significantly lower *v*_*0*_, A_line_ and hit distance were found after both training protocols (*p* ≤ 0.008), with the LLB protocol revealing a higher fatigue compared with the HLT protocol. However, the change of L-V relationship variables and softball-specific performance metrics (0.15 ≤ ES ≤ 1.05) were not as sensitive as that of blood lactate and RPE (1.30 ≤ ES ≤ 1.78).

**Discussion:**

Hence, changes in mechanical performance could be applied as a supplementary monitoring tool to be integrated into athletes’ daily routines, but should not be considered replacements for traditional fatigue indicators.

## Introduction

Softball is a widely practiced sport, involving batting and fielding between two teams on a large diamond-shaped field (Flyger et al., 2006). The efficacy of throwing and hitting is critical to achieving optimal performance and significantly influences the game outcome (Till et al., 2011; Kidokoro and Morishita, 2021; Stapleton et al., 2021). Throwing is a fundamental defensive skill in softball, enabling players to deliver the ball quickly and accurately to bases or teammates, preventing opponents from advancing or scoring (Chang et al., 2014). Hitting is the cornerstone of offensive play in softball, as it refers to players striking the ball with a bat to advance runners on base and ultimately score runs for their team (Kidokoro and Morishita, 2021). Biomechanically, the hit requires direct upper-body power application to drive bat acceleration, while the throw functions as a kinetic chain sequence where the lower body generates force that the upper body subsequently transmits (Oliver, 2014; Kidokoro and Morishita, 2021). These two crucial movements involve high velocities and extensive ranges of motion in the dominant arm generating significant distraction forces (i.e., forces directed away from the shoulder along the length of the upper arm) (Friesen et al., 2022). This can lead to shoulder-related injuries, posing considerable challenges for coaches and athletes by affecting an athlete’s performance and availability for competitions (Paul et al., 2021; Minetos et al., 2023). For example, a systematic review reported a mean rate of 4.01 shoulder injuries per 10,000 athlete-exposures in softball (Como et al., 2025). In addition to refining the throwing and hitting techniques, strengthening the upper body muscles is a highly effective strategy for improving sports performance and preventing injuries since it increases stability of the injury-prone area (Prokopy et al., 2008; Till et al., 2011). The packed schedules of competitive softball athletes often force the integration of strength training before technical training, which proximity creates a high risk that residual fatigue from the bench press might impair the quality and safety of subsequent hitting and throwing practice, highlighting the critical need for effective fatigue monitoring.

The bench press is one of the most commonly used exercises for strengthening the shoulder area, thanks to its widespread availability, simplicity, and functional relevance (Prokopy et al., 2008; Negrete et al., 2011). Evidence suggests that incorporating bench press exercises into training programs for overhead athletes significantly enhances performance metrics, including increased hitting power in baseball players (Miyaguchi and Demura, 2012), improved hitting distance in cricket athletes (Taliep et al., 2010) and enhanced throwing performance in both softball and baseball players (McEvoy and Newton, 1998). Moreover, shoulder strength is recognized as a pivotal factor in hitting and throwing success, as it not only reinforces shoulder stability but also enables greater acceleration of the throwing arm (Suzuki et al., 2024). However, careful consideration must be given to resistance training (RT) programming, as inappropriate intensities or excessive training volumes can lead to elevated fatigue levels, decreasing performance, which are associated with a higher risk of injury during bench press training (Small et al., 2010). To optimize the benefits of bench press for strength development while minimizing injury risk, it is imperative to closely monitor and regulate fatigue levels during training sessions.

Monitoring fatigue is crucial not only for injury prevention but also because it significantly influences athletes’ performance in technical and tactical training sessions following resistance workouts (Linnanio et al., 1998; Nájera-Ferrer et al., 2021; Sabag et al., 2021; Janicijevic et al., 2024). Common methods for monitoring fatigue during RT include evaluating the rating of perceived exertion (RPE), blood lactate concentration, and velocity loss (VL) (Sánchez-Medina and González-Badillo, 2011; Vieira et al., 2022). These methods have gained considerable popularity due to their positive correlation with key RT variables. For instance, RT volume and intensity have been positively correlated with RPE, blood lactate levels, and VL (Lantis et al., 2017; Pareja-Blanco et al., 2020; Jukic et al., 2022). However, each of these methods has limitations that warrant attention. For example, RPE is a subjective measure and can be influenced by the participant’s familiarity with the scale. Blood lactate monitoring is invasive, costly, and may not directly reflect changes in muscle dynamics (Vieira et al., 2022). Finally, VL monitoring is an objective and non-invasive measure, but it reflects fatigue for a particular set, making it less suitable for comparing fatigue responses across an entire training session (Pareja-Blanco et al., 2020; Li Z. et al., 2025). Furthermore, existing research on VL and fatigue has largely centered on male athletes. Comparative studies between sexes suggest that female athletes may require a greater degree of VL to elicit optimal training stimuli (Rissanen et al., 2022). Although female athletes have been shown to exhibit positive adaptive responses to VL-based training regimens (Zhang et al., 2023; Achermann et al., 2025), focused research efforts are warranted to establish evidence-based fatigue monitoring protocols tailored to female athletes. This is especially problematic for overhead athletes, where undetected fatigue accumulation can compromise throw and hit performance, and increase injury risk. To address these limitations, there is a need for a refined fatigue monitoring procedure of muscle function both before and after an RT session for female athletes.

A novel method introduced by García-Ramos et al. (2018b) utilizes the force-velocity (F-V) profile to assess the fatigue induced by a RT session. This methodology could reveal whether the fatigue protocol caused decrements in maximal force output, velocity output, or both, by assessing the intercept of the linear F-V relationship under both non-fatigued and fatigued conditions (García-Ramos, 2023; Li R. et al., 2025). A few studies already implemented this methodology for assessing selective fatigue effect of different training protocols. For example, heavy-load RT provoked the reduction in the muscle’s maximal force-generating capacity (*F*_*0*_), while squat jump protocols induced a higher reduction in the maximal velocity-generating capacity (*v*_*0*_) (Li et al., 2023). This demonstrates that different fatigue protocols can result in a decline in maximal force ability and maximal velocity ability occurring at different rates, rather than both declining proportionally, known as selective fatigue. Despite its merits, F-V modelling has limitations, including the requirement for specialized laboratory equipment to collect force data, which reduces its practical utility. Additionally, measurement points are often too distant from the intercept, potentially limiting the precision of the insights provided (Samozino et al., 2022; Pérez-Castilla et al., 2023). In contrast, the load-velocity (L-V) relationship offers a more practical alternative, requiring simpler measurements and providing improved inter-day reliability (Pérez-Castilla et al., 2021; 2023; Li Z. et al., 2025). The L-V relationship offers key metrics for fatigue monitoring: the load-axis intercept (*L*_*0*_) indicates maximal force capacity, the velocity-axis intercept (*v*_*0*_) represents maximal velocity ability, and the area under the L-V relationship line (A_line_) reflects the total power production potential (Pérez-Castilla et al., 2021; Li R. et al., 2025). Therefore, examining how the L-V relationship responds to varying loads and movement patterns offers valuable insights into its potential effectiveness for fatigue monitoring. This is particularly relevant to the athletes who often develop domain-specific fatigue toward either maximal force or velocity capacity following competition or training (Romero et al., 2022; Quidel-Catrilelbún et al., 2024).

This study aimed to address the identified research gaps by (I) investigating the feasibility of utilizing the L-V relationship variables alongside softball-specific performance metrics to monitor acute selective fatigue resulting from light-load ballistic (LLB) and heavy-load traditional (HLT) bench press training protocols; (II) whether there is selective fatigue between LLB and HLT protocols by examining differences in fatigue between light and heavy loads; and (III) evaluating whether this approach offers greater sensitivity compared to conventional methods such as RPE and blood lactate monitoring. We hypothesized that all the L-V relationship variables and softball-specific performance metrics would significantly decrease after the training protocols. Additionally, the LLB protocol was hypothesized to result in a greater decrement in light loads compared to high loads, whereas the opposite pattern was expected for the HLT protocol. Hence, the LLB protocol would preferentially decrease *v*_*0*_, while the HLT protocol would preferentially decrease *L*_*0*_. Additionally, we expected that the L-V relationship, combined with softball-specific performance metrics, would provide comparable sensitivity in detecting fatigue compared to traditional indicators like RPE and blood lactate.

## Methods

### Experimental approach to the problem

A crossover study design was employed to evaluate the feasibility of using the L-V relationship modelling and softball-specific performance metrics to monitor acute fatigue from different bench press training protocols, and to determine if this approach is more sensitive than traditional methods like RPE and blood lactate monitoring. Data collection occurred during the off-season, when participants followed light-intensity, softball-specific training routines while refraining from additional RT. The daily routine was the same for each session, comprised approximately 1 hour of combined throwing and hitting practice. Participants attended four testing sessions, each scheduled at the same time of day with at least 48-h intervals for recovery (García-Ramos et al., 2018b; Li et al., 2023). The first session involved anthropometric measurements, a one-repetition maximum (1RM) test, and familiarization with traditional and ballistic bench press variations performed at maximal intended velocity. The subsequent two sessions were conducted for each subject to assess the L-V relationships and softball-specific performance under three conditions: (I) control (no fatigue), (II) after a LLB fatigue protocol, and (III) following a HLT bench press fatigue protocol, with the LLB and HLT protocols performed in a randomized order *via* a computerized randomization algorithm. RPE and blood lactate levels were also measured during the LLB and HLT session to compare the sensitivity of traditional methods (RPE and blood lactate) with the newly proposed approaches for monitoring fatigue. All testing sessions were organized under similar environmental conditions.

### Participants

Considering an effect size of 0.5, an ɑ error probability of 0.05, a statistical power of 0.80, one group, three measurements, and assuming a correlation among repeated measures of 0.5, the sample size calculation indicated that at least 9 participants are required for detecting the hypothesized effects (G*Power software, version 3.1.9.6) (García-Ramos et al., 2018b). To minimize the risk of participant dropout, we conservatively recruited twelve female softball players from a provincial team in China who volunteered to participate in this study (age: 19.9 ± 1.2 years; body height:1.72 ± 0.04 m; body mass: 66.1 ± 7.9 kg; data presented as mean ± standard deviation [SD]). The cohort was intentionally restricted to female athletes to address the current lack of sex-specific data in this area and to ensure the findings are directly applicable to the population of elite female softball players. All participants were professional athletes with more than 3 years of softball competitive experience, and they had an average of 2 years of bench press training. None of the participants had recent injuries, and all attended testing sessions at the same time of the day without musculoskeletal pain that could negatively affect the study results. Prior to each testing session, all participants were verbally screened using a standardized protocol to confirm the absence of any current musculoskeletal pain or recent injuries (within the previous 48 h) that could adversely affect performance. All participants were informed about the research aims and procedures and gave their written consent to participate. All participants were highly familiar with performing bench press with maximal intended velocity. All participants completed all experimental sessions, and no data were excluded from the analysis. The study protocol adhered to the tenets of the Declaration of Helsinki and was approved by the Institutional Review Board of Beijing Sport University (2025345H).

### Procedures

**FIGURE 1:**
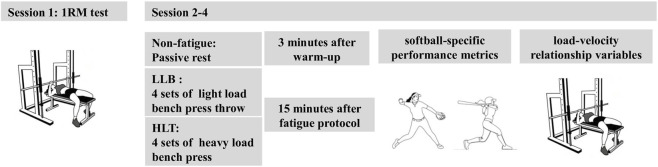
Schematic overview of the experimental design and testing protocol sequence.

Anthropometric measurements, 1RM assessment, and familiarization with traditional and ballistic bench press variations were conducted in the gym, while softball-specific performance measurements took place on the softball field. Participants attended all experimental sessions dressed in comfortable, softball-specific attire. During the first session, researchers measured participants’ body weight and height, followed by a bench press 1RM assessment conducted on a Smith machine using a standardized protocol (García-Ramos et al., 2018a). 1RM assessment was conducted under the supervision of an experienced spotter. Participants received standardized instructions and demonstrations to maintain proper lifting technique throughout the test with five-point body contact. The first session concluded with participants becoming familiarized with performing consecutive traditional and ballistic bench press variations at maximal intended velocity. The rest of the three experimental sessions involved distinct training protocols performed on separate days: (I) control, (II) LLB, and (III) HLT. Each session began with a general warm-up of 5 minutes of jogging and dynamic upper-limb stretches, followed by a specific warm-up with bench press throws at 20% and 40% of 1RM. When it comes to training protocols, the control condition involved no RT activity, LLB consisted of bench press throws at 30% of 1RM, while HLT comprised bench press exercises at 80% of 1RM. The traditional bench press maintained continuous barbell-hand contact, whereas the bench press throw featured a ballistic release, achieving complete barbell-hand separation at the terminal phase of the concentric movement. These loads were selected based on established training principles: 30% of 1RM typically maximizes power output in ballistic actions, whereas 80% of 1RM were widely applied for improving muscle hypertrophy and strength adaptations (Sarabia et al., 2017). Both LLB and HLT were performed at the maximal intended velocity and ended when the mean velocity fell below 80% of the MV from the fastest repetition of the given set. This 20% velocity loss criterion was selected to induce a significant level of neuromuscular fatigue, consistent with the study’s aim to examine selective fatigue effects (Jukic et al., 2022). Each training protocol included four sets with 3-min rests between them. Perceptual and biochemical measures were collected 5 minutes following the completion of each experimental protocol. RPE was recorded using the Borg CR10 scale, with a standard 1–10 scale applied (Weakley et al., 2019). Concurrently, blood lactate concentration was determined from a capillary blood sample drawn from the right earlobe and analyzed immediately with a validated portable lactate analyzer (Lactate Scout 4, EKF Diagnostics, Cardiff, United Kingdom) (Martos-Arregui et al., 2024). Softball-specific metrics (throw and hit distance) and bench press L-V relationship variables were assessed by three times: 3 minutes after warm-up in session 2 and 15 min after finishing the two training protocols (LLB and HLT) at session 3 and session 4 (Figure 1). Characteristics of the L-V relationship and sport-specific assessment are the following.

### Bench press load-velocity relationship modelling

Participants were required to perform the traditional bench press with maximal intended velocity for three repetitions at 20% and 40% 1RM, and two at 60% and 80% 1RM, with 3 min of rest between loads. This load protocol was widely used in the literature as it effectively captures the full spectrum of the relationship, from the high-velocity to the high-force domains (Miras-Moreno et al., 2023; Li R. et al., 2025). All participants were encouraged to lift the bar with maximal intended velocity and received the real-time velocity feedback in the form of an auditory prompt throughout all repetitions. The velocity of the barbell was measured with a linear position transducer (GymAware PowerTool, Kinetic Performance Technologies, Canberra, Australia). The MV was calculated as the mean velocity from the beginning of the concentric phase until the load reached its maximum height. The fastest MV for each load was recorded to establish the L-V relationship. A least-squares linear regression model, L(*v*) = *L*_*0*_ – *slope* × *v*, was applied to establish individualized L-V relationships, where *L*_*0*_ represents the theoretical load at zero velocity, and *slope* is the slope of the L-V relationship. The *v*_*0*_, representing the theoretical velocity at no external load, was calculated as *slope* divided by *L*_*0*_. The A_line_ was calculated as *L*_*0*_ × *v*_*0*_ × 0.5. The linear regressions used to establish the L-V relationships demonstrated excellent goodness-of-fit across all testing conditions, with *R* ^2^ values consistently exceeding 0.95 in both non-fatigue and fatigue states.

### Softball-specific performance metrics measurements

Participants were instructed to perform several practice hits and throws to prepare for the assessment. For the throwing test, participants used a standard softball to perform two overhand throws utilizing the crow-hop technique behind a line, throwing with maximum effort in order to achieve the longest throw distance possible. For the hit test, an iron pillar was positioned at home plate, with a standard softball placed atop the pillar. The iron pillar was positioned vertically at the center of home plate, with its height adjusted for each participant to be level with the iliac crest. Throwing and hitting distance was measured from the throwing line to the first point of ball contact with the ground using a standard measuring tape. Participants were instructed to hit the ball with maximum effort to achieve the greatest possible distance two times, and the distance was recorded by the primary investigator. Participants were permitted 2–3 unrecorded practice trials for familiarization prior to the formal testing. The greatest hit and throw distances were recorded as the final score. The rest time between attempts was set within 2 minutes. The within session reliability of throwing and hitting distance was verified in the non-fatigue session (CV ≤ 6 0.07, ICC ≥0 0.885).

### Statistical analyses

Descriptive data are expressed as means ± SD. The normality of the variables was confirmed using the Shapiro–Wilk test (P > 0.05). The sphericity assumption was using Mauchly’s test (all p > 0.05). Two-way repeated measures ANOVAs were conducted to compare the mechanical output variables (Repetition, maximal set MV, minimal set MV, and average set MV) across sets (Set 1, Set 2, Set 3, and Set 4) for different training protocols (LLB vs. HLT). One-way repeated measures ANOVAs with Bonferroni *post hoc* corrections were employed to compare L-V relationship variables (*L*_*0*_, *v*_*0*_, A_line_) and softball-specific metrics (throw and hit distances) among different training protocols (non-fatigue vs. LLB vs. HLT). Paired-sample t-test or Wilcoxon Signed-Rank test with Cohen’s d effect sizes (ES) were conducted to assess differences in RPE and blood lactate between two training protocols, separately. A two-way repeated measures ANOVAs with Bonferroni corrections *post hoc* was conducted to examine the effect of the training protocol (non-fatigue vs. LLB vs. HLT) and load (20%, 40%, 60%, and 80% 1RM) on barbell MV. The effects of fatigue on barbell velocity, L-V relationship variables, and softball-specific performance metrics between different conditions were quantified using Cohen’s d ES and percentage change. The criteria for interpreting the magnitude of the ES were: trivial (<0.20), small (0.20–0.59), moderate (0.60–1.19), large (1.20–2.00), and extremely large (>2.00). All statistical analyses were performed using SPSS software version 22.0 (SPSS Inc., Chicago, IL, United States) and statistical significance was set at an alpha level of 0.05 or less.

## Results

The LLB training protocol exhibited a faster maximal, minimal and average set MV, compared to the HLT protocol (F ≥ 466.8, p < 0.001). Additionally, it resulted in a greater number of repetitions, averaging 50.9 repetitions (LLB) versus 25 repetitions (HLT), with approximately 6.5 additional repetitions per set (F = 42.3, p < 0.001). All the mechanical output variables decreased with increasing number of set (F ≥ 8.5, p ≤ 0.015) (Table 1).

**TABLE 1:** Comparison of repetitions performed, maximal, minimal and average set mean velocity between light-load ballistic and heavy-load traditional training protocols.

Variables	Training protocol	Set number				ANOVA		
		Set 1	Set 2	Set 3	Set 4	Protocol	Set	Interaction
Repetitions (n)	LLB	14.0 ± 3.7	12.0 ± 4.1	13.2 ± 3.6	11.7 ± 2.2	**F = 42.3**	**F = 8.5**	F = 2.5
	HLT	5.9 ± 2.5	6.4 ± 2.0	6.3 ± 2.6	6.4 ± 2.3	**p < 0.001**	**p = 0.015**	p = 0.083
Maximal set MV (m·s^-1^)	LLB	1.27 ± 0.08	1.17 ± 0.09	1.14 ± 0.05	1.11 ± 0.08	**F = 536.0**	**F = 28.4**	**F = 5.5**
	HLT	0.45 ± 0.06	0.44 ± 0.05	0.41 ± 0.06	0.40 ± 0.06	**p < 0.001**	**p < 0.001**	**p = 0.004**
Minimal set MV (m·s^-1^)	LLB	0.97 ± 0.07	0.89 ± 0.07	0.87 ± 0.09	0.83 ± 0.07	**F = 466.8**	**F = 16.6**	F = 2.1
	HLT	0.33 ± 0.05	0.31 ± 0.06	0.30 ± 0.07	0.29 ± 0.06	**p < 0.001**	**p < 0.001**	p = 0.115
Average set MV (m·s^-1^)	LLB	1.08 ± 0.07	0.99 ± 0.07	0.97 ± 0.05	0.95 ± 0.06	**F = 1224.9**	**F = 10.6**	**F = 6.8**
	HLT	0.37 ± 0.06	0.37 ± 0.06	0.33 ± 0.05	0.31 ± 0.05	**p < 0.001**	**p < 0.001**	**p = 0.001**

MV, mean velocity; LLB, light-load ballistic; HLT, heavy-load traditional.

Significant differences are emphasized in bold.

**FIGURE 2:**
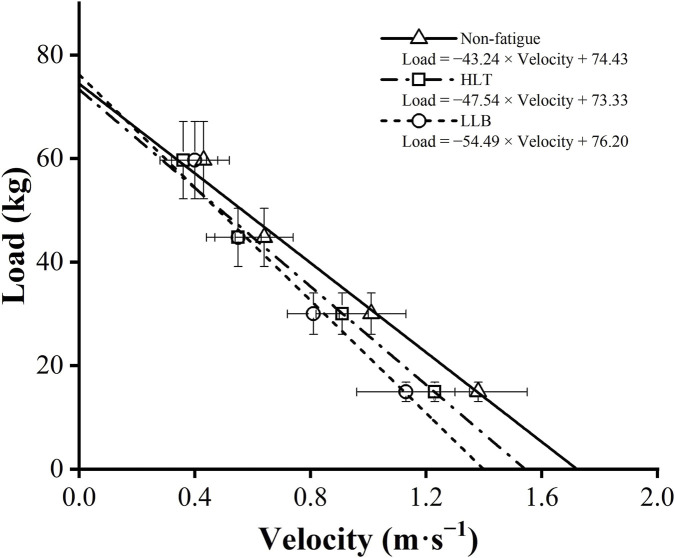
Differences in the load-velocity relationships between non-fatigue (triangles and solid line), HLT (squares and dash dot line), and LLB (circles and short dash line) conditions.

**FIGURE 3:**
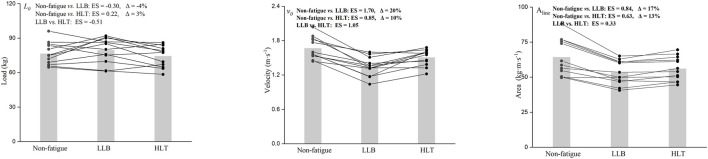
Comparison of individual changes in L-V relationship variables (load-axis intercept [left panel; *L*_*0*_]; velocity-axis intercept [middle panel; *v*_*0*_]; area under the line; [right panel; A_line_]) between different training protocols (non-fatigue, light-load ballistic [LLB] and heavy-load traditional [HLT]). Significant differences are emphasized in bold.

Significant main effect of the training protocol was found for *v*_*0*_ (F = 28.2, p < 0.001), and A_line_ (F = 26.3, p < 0.001), but not for *L*_*0*_ (F = 2.8, p = 0.084). When compared to the non-fatigue protocol, both training protocols led to moderate reductions in A_line_ (LLB: ES = 0.84, Δ = 17%; HLT: ES = 0.62, Δ = 10%), with the LLB protocol causing a greater reduction primarily due to a more substantial reductions in *v*_*0*_ (LLB: ES = 1.70, Δ = 20%; HLT: ES = 0.85, Δ = 10%) rather than *L*_*0*_ (LLB: ES = −0.30, Δ = −4%; HLT: ES = 0.22, Δ = 3%) (Figures 2, 3). When comparing protocols, the LLB protocol resulted in a significantly higher reductions in *v*_*0*_ (ES = 1.05) and A_line_ (ES = 0.33) compared with the HLT protocol.

**FIGURE 4:**
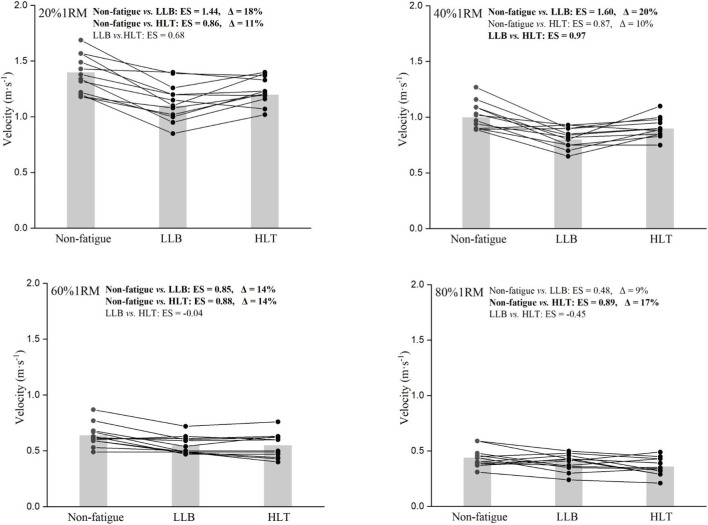
Comparison of individual changes in mean velocity at 20%1RM (upper-left panel), 40%1RM (upper-right panel), 60%1RM (lower-left panel), and 80%1RM (lower-right panel) between different training protocols (non-fatigue, light-load ballistic [LLB], and heavy-load traditional [HLT]).

Significant effects of the training protocol (F = 34.5, p < 0.001), load (F = 266.3, p < 0.001), as well as their interaction (F = 6.3, p < 0.001) were observed for MV at individual loads. The LLB protocol caused a large reduction in MV under light loads (20%1RM: ES = 1.44, Δ = 18%; and 40%1RM: ES = 1.60, Δ = 20%) and a moderate to small reduction under heavier loads (60%1RM: ES = 0.85, Δ = 14%; and 80%1RM: ES = 0.48, Δ = 11%). The HLT protocol caused moderate reductions in MV across all loads (0.86 ≤ ES ≤ 0.89, 10% ≤ Δ ≤ 17%). Pairwise comparisons indicated significant effects for all comparisons at training protocol and loads main effects. The pairwise comparisons of MV at different training protocols were presented in Figure 4.

**FIGURE 5:**
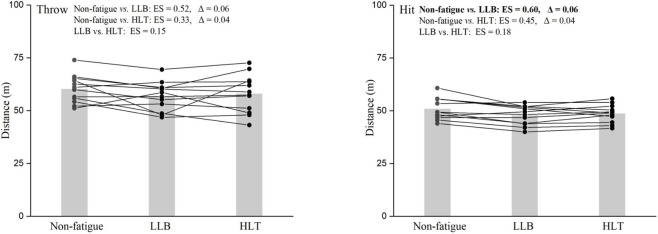
Comparison of individual changes in softball-specific performance metrics (throw distance [left panel], hit distance [right panel]) between different training protocols (non-fatigue, light-load ballistic [LLB] and heavy-load traditional [HLT]). Significant differences are emphasized in bold.

For the softball-specific performance metrics, there was a significant reduction in hit distance (F = 6.1, p = 0.008) but not in throw distance (F = 2.2, p = 0.130) following both training protocol, with both metrics showing small reductions (0.33 ≤ ES ≤ 0.60; 4% ≤ Δ ≤ 6%). Pairwise comparisons are presented in Figure 5.

Both training protocols resulted in large and significant pre-to-post increases in RPE (Z = 3.0, p = 0.003) and blood lactate levels (t = 3.5, p = 0.005). When comparing the post-training fatigue indicator levels, a higher level of RPE (ES = 1.78) and blood lactate (ES = 1.30) was observed for the LLB protocol compared with the HLT protocol, whereas the L-V relationship variables (0.33 ≤ ES ≤ 1.05) and softball specific-performance metrics (0.15 ≤ ES ≤ 0.18) showed trivial to moderate differences between the two training protocols (Table 2).

**TABLE 2:** Comparison of the fatigue indicators, load-velocity relationship variables and softball-specific performance metrics under different protocols.

Parameter	Non	LLB	HLT	ES (LLB vs. Non)	ES (HLT vs. Non)	ES (LLB vs. HLT)
*L*_0_ (kg)	76.7 ± 9.7	79.6 ± 10.9	74.5 ± 9.4	−0.30 (−0.89, 0.27)	0.22 (−0.36, 0.81)	−0.51 (−1.32, 0.31)
*v*_0_ (m·s^-1^)	1.67 ± 0.19	1.35 ± 0.17	1.5 ± 0.14	1.70 (0.68, 2.72)	0.85 (0.16, 1.55)	1.05 (0.19, 1.91)
A_line_ (kg·m·s^-1^)	64.2 ± 13.2	53.4 ± 8.4	56.1 ± 8.5	0.84 (−0.07, 1.74)	0.63 (−0.12, 1.38)	0.33 (−0.48, 1.13)
Throw (m)	60.3 ± 6.6	56.9 ± 6.8	58.1 ± 9.0	0.52 (−0.09, 1.13)	0.33 (−0.27, 0.93)	0.15 (−0.65, 0.96)
Hit (m)	51.0 ± 5.1	47.9 ± 4.5	48.7 ± 4.2	0.60 (−0.10, 1.30)	0.45 (−0.17, 1.06)	0.18 (−0.63, 0.98)
RPE (score)	—	5.3 ± 1.3	3.5 ± 0.5	—	—	1.78 (0.92, 2.63)
Lactate (mmol·L^-1^)	—	5.8 ± 2.4	3.4 ± 0.9	—	—	1.30 (0.36, 2.28)

ES, effect size; LLB, light-load ballistic; HLT, heavy-load traditional; *L*_0_, load-intercept; *v*_0_, velocity-intercept; A_line_, area under the line.

## Discussion

This study aimed to investigate the applicability of L-V relationship variables and softball-specific performance metrics for monitoring the selective fatigue effects of HLT and LLB bench press training protocols. The main finding supported the application of L-V relationship variables and softball-specific as a complementary tool in monitoring fatigue instead of replacing the traditional methods. The main results revealed significant decrements in *v*_*0*_, A_line_, and hit distance following both training protocols. Specifically, the LLB training protocol induced higher *v*_*0*_ and A_line_ decrements compared to the HLT protocol, characterized by the higher repetitions performed and faster velocity produced. Moreover, the L-V relationship variables and softball-specific performance metrics revealed smaller differences between the two training protocols compared to traditional fatigue indicators including blood lactate and RPE. Collectively, these findings suggest that L-V relationship variables and softball-specific performance metrics could be utilized to monitor resistance training-induced fatigue. However, their effectiveness in distinguishing the magnitude of fatigue between different protocols may be limited.

We noted that *v*_*0*_ and A_line_ were more sensitive than *L*_*0*_ in detecting the fatigue, with *v*_*0*_ emerging as the most sensitive parameter by showing the most pronounced deterioration following the LLB protocol compared to the HLT protocol. This contrasted with previous studies suggesting maximal power output (A_line_ in L-V relationship variables) as the preferred metric for monitoring acute fatigue (Jones, 2010; García-Ramos et al., 2018b). The observed discrepancy might stem from the fundamental methodological difference between F-V and L-V relationships. Whereas the F-V relationship modeled force and velocity outputs at given loads, which typically declined concurrently under fatigue conditions, the L-V relationship variables only revealed velocity declines across submaximal loads with no actual change in the absolute loads themselves. Additionally, the LLB protocol applied ballistic exercise with higher movement velocity and involved more than twice the number of repetitions compared to HLT, further exacerbating the greater decline in *v*_*0*_ compared to that of the HLT protocol. This aligned with former studies which suggested that the muscle’s ability to produce maximal velocity was more sensitive to a greater number of repetitions performed and higher movement velocity (García-Ramos et al., 2018b; Şentürk et al., 2024). This increased repetition volume likely resulted in greater accumulated fatigue, as reflected in the higher RPE and blood lactate measurements. The LLB protocol predominantly induced fatigue in *v*_*0*_, which was underpinned by potential mechanisms including impaired high-frequency neural drive and reduced recruitment of fast-twitch fibers (Hill et al., 2022). Therefore, *v*_*0*_ should be integrated into the daily monitoring framework as a key metric during velocity-oriented training phases.

In line with previous research, the training protocol exhibited selective effects on the MV at different loads (García-Ramos et al., 2018b). Specifically, more pronounced reductions in MV at 60% 1RM (Δ = 14%) and 80% 1RM (Δ = 17%) were observed following the HLT protocol, whereas only approximately 10% reductions were noted at 20% 1RM and 40% 1RM. Conversely, the LLB protocol had a more substantial impact on MV at lighter loads compared to heavier ones, with larger reductions observed at 20%1RM (Δ = 18%) and 40%1RM (Δ = 20%) compared to 60%1RM (Δ = 14%) and 80%1RM (Δ = 9%). Of note is that the L-V relationship variables were measured using the traditional bench press, whereas the training protocols employed the bench press throw, which elicited greater velocity and power outputs. This likely explained why the velocity decrease at 40% 1RM was greater than at 20% 1RM in the LLB protocol. However, this remained a speculative interpretation and required verification in future studies designed specifically to examine neuromuscular specificity. Another potential reason could be variations in the scheduling of fatigue assessments. For example, Pálinkás et al. (2024) tested the F-V relationship 24 h after HLT squat protocol and found that muscle ability to produce maximal velocity was affected, whereas maximal force remained largely unchanged. However, when measured immediately following HLT protocol, the decline in maximal force would likely be more pronounced than that in maximal velocity (Li et al., 2023). The interplay of fatigue and enhancement led to selective effects that manifested in different directions at different time points.

The differing magnitudes of MV decline across loads may help explain the light increase in *L*_*0*_ following the LLB protocol, despite a general decline in barbell velocity across all loads. The rise in *L*_*0*_ might have been influenced by the increase in the slope of the L-V relationship, as the heaviest load measured accounted for only around 70% of *L*_*0*_. Similar increments in intercept after fatigue and trivial minimal changes at certain endpoints have been reported in previous studies (González-Hernández et al., 2023). However, they did not compare the changes in L-V relationship variables under conditions closer to the endpoint, such as loads exceeding 90% or even at 1RM. Thus, it remained unclear whether the increase in *L*_*0*_ resulted from overestimation due to changes in slope or if selective fatigue led to an improvement in the muscle’s ability to generate maximal force. To give insights to this question, it is important that future studies also assess L-V relationship variables with higher load or even the maximal isometric force.

For the softball-specific performance metrics, we observed a significant decrement after training in hit distance, but not in throw distance. These findings are different from the results of Nuño et al. (2016), which indicated that a circuit training protocol significantly affected the kinematics of throwing performance of handball players. Decline in throwing velocity was also observed after a running protocol in baseball players (Freeston et al., 2014). The differences in fatigue effects observed in our study compared to those of Nuño et al. (2016), Freeston et al. (2014) might be attributed to the distinct muscle groups involved in the softball throw versus handball and baseball throws. Specifically, throw performance in softball relied to a great extent on lower limb muscles due to the acceleration provided by the approach run compared to handball and baseball throw (Oliver, 2014). In contrast, hitting performance in softball engages predominantly upper limb muscles. Given that the training protocol in our study only involved the bench press, it is expected to have a greater impact on sport-specific activities that primarily engage upper-body muscles (Oliver, 2014). The divergent findings regarding throwing may reflect fundamental methodological differences: our study focused on isolated upper-body fatigue, whereas previous studies used a whole-body approach. Overall, sport-specific performance metrics could be used to monitor fatigue, however, the selection of appropriate fatigue monitoring indicators remained a topic worthy of discussion. Despite statistical significance observed in some softball-specific performance metrics, their trivial effect sizes raise questions about their practical utility for making definitive training adjustments. Furthermore, the sport-specific nature of throwing mechanics limits the generalizability of these findings. Therefore, caution was warranted when generalizing these findings to sports with different movement patterns.

Although the alteration of kinetic and kinematic variables has recently been proposed as an alternative to traditional fatigue indicators (Jones, 2010; Amatori et al., 2020), our findings suggested that the sensitivity of L-V relationship variables and softball-specific performance metrics was less pronounced compared to traditional fatigue indicators to distinguish between the two training protocols.

Specifically, the traditional indicators showed large effect sizes (ES ≥ 1.30) for distinguishing between protocols, while L-V variables and softball-specific performance metrics showed only small to moderate effect sizes (ES ≤ 1.05). The difference in the hit and throw distances between the two training protocols was trivial, insufficient to clearly reveal the distinct fatigue effects of the two training protocols. Additionally, A_line_ and *v*_*0*_, which were more sensitive in the training protocol setups, both showed only small to moderate differences between the two training protocols. The lower sensitivity of L-V variables compared to traditional indicators may be attributed to several factors, such as the fact that the 15-min post-protocol assessment probably captured a phase of partial neuromuscular recovery. Considering these findings, while changes in L-V relationship variables and softball-specific performance metrics reflected neuromuscular fatigue, they were less effective than traditional fatigue indicators for comparing fatigue levels across protocols.

Finally, a number of limitations should be acknowledged. Firstly, the non-fatigue and fatigued L-V relationship variables and softball-specific performance metrics were tested on separate days. Due to the potential between-day variability, the test results might not entirely capture the fluctuations in fatigue levels (Li et al., 2023; Pérez-Castilla et al., 2023). Secondly, we tested L-V relationship variables in a smith machine and softball-specific metrics approximately 15 min after finishing the last set of training protocols. Although our training volume was sufficiently large and the duration was long enough, theoretically, it should not have resulted in delayed effects that could improve performance (Blazevich and Babault, 2019; Yuan et al., 2023). However, we were unable to determine whether the fatigue level was consistent across all time points following the training protocols such as immediately post-exercise. Future studies should compare different time points (post-set, or at various time points after completing the entire protocol) to identify the most sensitive measurement time. Thirdly, the generalizability of our findings is limited by the exclusive focus on sport-specific metrics in female softball athletes. While this study has approved the hit distance as a fatigue monitoring tool. Future research should investigate the utility of sport-specific task monitoring in other athletic disciplines to determine its broader applicability, as well as in different athlete populations including males and youth cohorts. Fourthly, the *post hoc* analysis revealed that our sample size of 12 participants was insufficient to provide a reliable estimate of the effect size for some variables such as *L*_*0*_ and throw distance. The non-significant results for variables such as *L*_*0*_ and throw distance should not be interpreted as evidence of no effect, but as findings that are inconclusive and require verification in a larger study. Lastly, the present study only examined acute fatigue responses. Future investigations should use longitudinal tracking designs to examine how these monitoring variables evolve throughout a complete training season, and how they are related to long-term athletic performance and injury risk. Future research should also establish individualized thresholds for meaningful changes in L-V relationship variables to enhance the practical utility of this monitoring approach for guiding training decisions in athletic populations.

## Conclusion

Within the given sample size and cross-sectional nature of our research, we found that L-V relationship variables and softball-specific performance metrics could effectively monitor the selective effects of HLT and LLB bench press training protocols in female softball athletes. The LLB protocol resulted in greater reductions in *v*_*0*_, A_line_, and hitting performance, indicating a primary impairment in velocity-oriented capacities due to its higher velocity output and more training volume, whereas the HLT protocol produced greater decrements in maximal force output. These findings should be interpreted considering that assessments were conducted 15 min post-exercise, which may have allowed partial recovery and influenced the sensitivity of mechanical performance measures. However, when compared with traditional indicators such as RPE and blood lactate, L-V parameters and softball-specific performance metrics demonstrated lower sensitivity in distinguishing between training protocols, as evidenced by the trivial-to-moderate effect sizes for the differences between the two fatigue protocols. Collectively, these findings suggested that L-V relationship variables and softball-specific performance metrics represented a field-applicable approach for monitoring acute fatigue in female athletes. The fatigue status of athletes could be determined by comparing post-training measurements against their individual baseline or daily pre-training values. Decreases in L-V relationship variables and softball-specific performance metrics enabled the precise identification of fatigue-induced impairments to specific neuromuscular functions. Coaches and sports scientists should use L-V relationship monitoring to supplement rather than replace traditional fatigue indicators like RPE and blood lactate, particularly when comparing fatigue across different training protocols in practical settings.

## Data Availability

The raw data supporting the conclusions of this article will be made available by the authors, without undue reservation.
